# Supplementary material for: Patterns of Intron Gain and Loss in Fungi
Source: PLoS Biol. 2004 Nov 30;2(12):e422. doi: 10.1371/journal.pbio.0020422 (PMC532390; doi:10.1371/journal.pbio.0020422)
Supplement: Table S1 — Also available at http://genes.mit.edu/NielsenEtAl/. (4.3 MB ZIP). [file pbio.0020422.st001.zip › NielsenEtAl/html/1189.html]

AN2142.1.NCU01249.1.MG07060.1.FG07141.1


```
 CLUSTAL W (1.82) Multiple Sequence Alignments - Introns Inserted


Sequence 1: MG07060.1	551 aa
Sequence 2: FG07141.1	552 aa
Sequence 3: NCU01249.1	548 aa
Sequence 4: AN2142.1	553 aa
Alignment Length: 556 aa
Number Identitical Residues: 430 aa
Alignment Score (without introns) 17625


MG07060.1 	MAERYIPEHRRTQFKAKNTFKPEELRRRREEQQVEIRKAKREENLAKRRGIGTGADRPGA
NCU01249.1	MADRYIPEHRRTQFKAKSAFKPDELRRRREEQQVEIRKAKREENLAKRRGIGAGDSRPGA
FG07141.1 	MAERYIPEHRRTQFKAKNTFKPEELRRRREEQQVEIRKAKREENLAKRRGIGTGENRPGA
AN2142.1  	MAERYIPEHRRTQYKARNQFRPDELRRRREEQQVEIRKQKREENLAKRRGIQTRDGGIGV
          	**:**********:**:. *:*:*************** ************ :  .  *.

MG07060.1 	SLG-AAPDSDDDTAPTESQ0LNEDLPQMVQGVFSESIDAQIQATTKFRKLLSKERNPPIE
NCU01249.1	SLG-AAPDSDDENPPTESQ0LSEDLPKMVEGVFSSEIDKQIQATTKFRKLLSKERNPPIE
FG07141.1 	SLG-AAPDSDDESAPTESQ0LNEDLPQMVQGVFSDQIDSQIQATTKFRKLLSKERNPPIE
AN2142.1  	GGGMAAAESDDEASAIESE0LNVELPEMVKGVFSDQIEAQIQATTKFRKLLSKERNPPIE
          	. * **.:***: .. **: *. :**:**:****..*: *********************

MG07060.1 	EVIKTGVVSRFVEFLRSPHTLVQFEAAWALTNIASGSATQTQVVIEAGAVPIFVELLGSP
NCU01249.1	EVIKTGVVGRFVEFLRSPHTLVQFEAAWALTNIASGSATQTQVVIEAGAVPIFVELLGSP
FG07141.1 	EVIKTGVVSRFVEFLRSPHTLVQFEAAWALTNIASGSATQTQVVIEAGAVPIFVELLASP
AN2142.1  	RVIETGVVSRFVEFLRSPHTLVQFEAAWALTNIASGSAQQTQVVIEAGAVPIFVELLSSP
          	.**:****.***************************** ******************.**

MG07060.1 	EPDVREQAVWALGNIAGDSPQCRDYVLSCGALKPLLNLLGDSRKLSMLRNATWTLSNFCR
NCU01249.1	EPDVREQAVWALGNIAGDSPQCRDYVLSCGALRPLLTLLGDSRKLSMLRNATWTLSNFCR
FG07141.1 	EPDVREQAVWALGNIAGDSPHCRDYVLSCGALKPLLNLLGDSRKLSMLRNATWTLSNFCR
AN2142.1  	EPDVREQAVWALGNIAGDSPQCRDFVLNAGALRPLLTLINDGRKISMLRNATWTLSNFCR
          	********************:***:**..***:***.*:.*.**:***************

MG07060.1 	GKTPQPDWTT0IAPALPVLAKLVYSLDDEVLIDACWAISYLSDGSNDKIQAVIEAGIPRR
NCU01249.1	GKTPQPDWNT0IAPALPVLAKLVYSLDDEVLIDACWAISYLSDGSNDKIQAVIEAGIPRR
FG07141.1 	GKTPQPDWTT0IAPALPVLSKLVYSLDDEVLIDACWAISYLSDGSNDKIQAVIEAGIPRR
AN2142.1  	GKTPQPDWNT0IAPALPVLAKLIYMLDDEVLIDACWAISYLSDGPNEKIQAVIEAGIPRR
          	********.* ********:**:* *******************.*:*************

MG07060.1 	LVELLMHASTSVQTPALRSVGNIVTGDDVQTQVIINCGALPCLLSLLGSNKDGIRKEACW
NCU01249.1	LVELLMHASTSVQTPALRSVGNIVTGDDVQTQVIINCGALPCLLSLLSSNKDGIRKEACW
FG07141.1 	LVELLMHASTSVQTPALRSVGNIVTGDDVQTQVIINCGALPCLLSLLSSTKDGIRKEACW
AN2142.1  	LVELLMHASTSVQTPALRSVGNIVTGDDVQTQVIINCGALPALLSLLSSTKDGIRKEACW
          	*****************************************.*****.*.**********

MG07060.1 	TISNITAGNSAQIQSVVDANIIPPLIHLLSNGDLKTRKEACWAISNATSGGLQKPDQIRY
NCU01249.1	TISNITAGNSAQIQSVIDANIIPPLIHLLSHADLKTRKEACWAISNATSGGLQKPDQIRY
FG07141.1 	TISNITAGNSSQIQAVIDANIVPPLIHLLQNGDLKTRKEACWAISNATSGGLQKPEQIRY
AN2142.1  	TISNITAGNSSQIQSVIDAGIIPPLVHLLANGDFKTRKEACWAISNATSGGLQKPDQIRY
          	**********:***:*:**.*:***:*** :.*:*********************:****

MG07060.1 	LVAQGCIKPLCDLLSCPDNKIIQVALDGLENILKVGDLDKQAAGEGQDSINRYALFIEEC
NCU01249.1	LVAQGCIKPLCDLLACPDNKIIQVALDGLENILKVGELDKNAAGDGPDSINRYALFIEEC
FG07141.1 	LVAQGCIKPLCDLLGCPDNKIIQVALDGLENILKIGDLDKQAAGESGDSINRYALFIEEC
AN2142.1  	LVTQGCIKPLCDLLACPDNKIIQVALDGLENILKVGEMDKEAG-QGDAHVNRYALFIEEA
          	**:***********.*******************:*::**:*. :.   :*********.

MG07060.1 	GGMEKIHECQTNANEEIYMKAFNIIEKYFSDDDENADDAAPAQ--GPNGTFGFGNPSGGQ
NCU01249.1	GGMEKIHDCQTNANEEIYMKAYNIIEKYFSDEDEAGDEAMGAQ-----QQFGFG-ASGGA
FG07141.1 	GGMEKIHDCQNNANEEIYMKAYNIIEKYFSDDEENADDGMAQQPAGPNGTFGFG-TNGAA
AN2142.1  	GGMEKIHDCQNNANEEIYMKAYNIIEKYFSDEDEAAGDIDELAP--QQTQTGFTLGATQQ
          	*******:**.**********:*********::* ..:     .   .   **       

MG07060.1 	QQNQFSF--ANGGESMDM
NCU01249.1	QQGGFNFG-ANGTESMDM
FG07141.1 	PAGGFDF--AKGNDAMDM
AN2142.1  	QPGGFSFGGANGGDSMDM
          	  . *.*..*:* ::***
```
